# Functional Specificity of Cardiolipin Synthase Revealed by the Identification of a Cardiolipin Synthase CrCLS1 in *Chlamydomonas reinhardtii*

**DOI:** 10.3389/fmicb.2015.01542

**Published:** 2016-01-12

**Authors:** Chun-Hsien Hung, Koichi Kobayashi, Hajime Wada, Yuki Nakamura

**Affiliations:** ^1^Institute of Plant and Microbial Biology, Academia SinicaTaipei, Taiwan; ^2^Department of Life Sciences, Graduate School of Arts and Sciences, The University of TokyoTokyo, Japan; ^3^Japan Science and Technology Agency, CRESTSaitama, Japan; ^4^Japan Science and Technology Agency, PRESTOSaitama, Japan

**Keywords:** CDP-alcohol phosphotransferase, cardiolipin, cardiolipin synthase, phosphatidylglycerol, PGPS, *Chlamydomonas reinhardtii*, Synechocystis sp. PCC 6803, *Saccharomyces cerevisiae*

## Abstract

Phosphatidylglycerol (PG) and cardiolipin (CL) are two essential classes of phospholipid in plants and algae. Phosphatidylglycerophosphate synthase (PGPS) and cardiolipin synthase (CLS) involved in the biosynthesis of PG and CL belong to CDP-alcohol phosphotransferase and share overall amino acid sequence homology. However, it remains elusive whether PGPS and CLS are functionally distinct *in vivo*. Here, we report identification of a gene encoding CLS in *Chlamydomonas reinhardtii, CrCLS1*, and its functional compatibility. Whereas *CrCLS1* did not complement the growth phenotype of a PGPS mutant of *Synechocystis* sp. PCC 6803, it rescued the temperature-sensitive growth phenotype, growth profile with different carbon sources, phospholipid composition and enzyme activity of *Δcrd1*, a CLS mutant of *Saccharomyces cerevisiae*. These results suggest that *CrCLS1* encodes a functional CLS of *C. reinhardtii* as the first identified algal CLS, whose enzyme function is distinct from that of PGPSs from *C. reinhardtii*. Comparison of CDP-alcohol phosphotransferase motif between PGPS and CLS among different species revealed a possible additional motif that might define the substrate specificity of these closely related enzymes.

## Introduction

Functional specificity of an enzyme is crucial in keeping metabolic reactions in order. This largely relies on the substrate specificity defined by the catalytic motif. Thus, enzymes are often categorized into groups according to the existence of common catalytic motif(s). In phospholipid metabolism, a number of important reaction steps are catalyzed by CDP-alcohol phosphotransferases (Li-Beisson et al., 2013, 2015). These include CLS, PGPS, phosphatidylinositol (PI) synthase, phosphatidylserine (PS) synthase, phosphatidylcholine (PC) synthase, and aminoalcohol phosphotransferase for the biosynthesis of CL, PG, PI, PS, PC, and PE, respectively. Because these are the major phospholipid classes found in diverse organisms from bacteria to mammals and seed plants, it can be stated that CDP-alcohol phosphotransferases are crucial in the entire phospholipid metabolism.

In plants and algae, PG is an indispensable phospholipid class in photosynthetic function (Hagio et al., 2000, 2002; Sato et al., 2000; Babiychuk et al., 2003; Yu and Benning, 2003). Moreover, CL, which is an anionic phospholipid class widely distributed in different kingdom and found exclusively at the inner membrane of mitochondria (Lewis and McElhaney, 2009), has an essential role in mitochondrial function and thus plant growth (Katayama et al., 2004; Pineau et al., 2013). The biosynthesis of these lipid classes begins with the conversion of phosphatidic acid (PA) into CDP-diacylglycerol (CDP-DAG) by CDP-DAG synthase (CDS; Sato et al., 2000; Haselier et al., 2010; Zhou et al., 2013). Next, PGPS coverts CDP-DAG to phosphatidylglycerol phosphate (PGP), which is dephosphorylated by PGP phosphatase (PGPP) to produce PG (Muller and Frentzen, 2001; Hagio et al., 2000, 2002; Wu et al., 2006; Osman et al., 2010; Hung et al., 2015b). Furthermore, PG is converted to CL by CLS in mitochondria (Kadenbach et al., 1982; Jiang et al., 1997, 1999). Initially, 3 PGPSs were proposed in *Arabidopsis thaliana* (PGP1, PGP2, and PGP3) based on the amino acid sequence similarity (Xu et al., 2002). However, the third isoform (PGP3) was later shown not to be a functional PGPS but instead functions as CLS (Katayama et al., 2004). Subsequent gene knockout studies defined distinct *in vivo* function of CLS associated with mitochondrial function (Pineau et al., 2013). Thus, PGPS and CLS are functionally independent, although they are homologous and belong to the same CDP-alcohol phosphotransferase family in *A. thaliana*. Recently, we identified and characterized genes for PGPS of *Chlamydomonas reinhardtii* (Hung et al., 2015a). We demonstrated two functional PGPS isoforms; however, genome-wide search identified an additional PGPS homolog, which is more homologous with CLS. This reminded us of the case in *A. thaliana* described above. Because reciprocal genetic complementation was not performed yet in *A. thaliana* or any other model organisms, it remains elusive whether PGPS and CLS are functionally distinct *in vivo*.

In this report, we identified the additional homolog of PGPS in *C. reinhardtii*, designated *CrCLS1* (*Cre13.g604700*), and performed reciprocal functional complementation assay using *pgsA*, a PGPS mutant of *Synechocystis* sp. PCC 6803 and Δ*crd1*, a CLS mutant of *Saccharomyces cerevisiae*. The result of functional complementation in these mutants, along with phenotype observation, lipid analysis and enzyme activity assay, demonstrated that *CrCLS1* encodes a functional CLS but not PGPS. We compared sequence similarity in detail between PGPS and CLS and noted some difference adjacent to the defined CDP-OH-P motif. Our results suggest non-overlapping function of PGPS and CLS, through the identification and characterization of CLS in *C. reinhardtii* as the first report of CLS in algae.

## Materials and Methods

### Strains

The strains produced in this work are listed in Supplementary Table S1.

### Protein Sequence Analysis

The multiple alignment of protein sequences was performed by use of CLUSTALW^1^. The mitochondrial targeting sequence was predicted by use of the subcellular localization program MitoProtII^2^ (Claros and Vincens, 1996).

### Cloning of Plasmid Vectors

*CrCLS1* (*Cre13.g604700*): To construct pCH069, a 1,060-bp fragment was amplified from the cDNA template of *C. reinhardtii* strain CC-503 (cw92 mt+) with the primers CH227 and CH228, and cloned into pENTR/D-TOPO. Then, to construct pCH178, the open reading frame (ORF) of *CrCLS1* was amplified from pCH069 with the primers CH831 and CH832, and inserted into *Xba*I and *Eco*RI sites of pCH078 (Hung et al., 2013). To construct pCH158, the ORF of *CrCLS1* was amplified from pCH069 with the primers CH776 and CH777 and inserted into *Nde*I and *Hpa*I sites of pTCP2031V (Satoh et al., 2001). The primers and plasmids used in this study are described in Supplementary Tables S2 and 3, respectively.

### Complementation Assay of the *Synechocystis* sp. PCC 6803 *pgsA* Mutant by *CrCLS1*

Complementation assay of the *Synechocystis* sp. PCC 6803 *pgsA* mutant by *CrCLS1* (pCH158) was performed as described previously (Hung et al., 2015a).

### Complementation Assay of the *S. cerevisiae Δcrd1* Mutant by *CrCLS1*

Complementation assay of the *S. cerevisiae Δcrd1* mutant by *CrCLS1* (pCH178) was performed as described previously (Hung et al., 2015a).

### Lipid Extraction and Analysis

Lipid extraction and analysis were performed as previously described (Hung et al., 2013) except that 2D thin-layer chromatography (TLC) was used to separate phospholipid classes with the solvent system of chloroform/methanol/7 N ammonia 120:80:8 (by vol) for the first dimension and chloroform/methanol/acetic acid/water 170:20:15:3 (by vol) for the second dimension (Nakamura et al., 2003).

### Radiolabeling Assay of CLS Activity

Logarithmically growing cells were resuspended in 5 ml SC-Ura medium at the cell density (OD_600_ of 5) with 30 μCi KH_2_^32^PO_4_ (PerkinElmer). After shaking incubation for 8 h at room temperature, lipids were extracted from cells by the method of Bligh and Dyer (1959). Lipids spotted on a TLC plate (Silica gel 60G, Merck) were developed with chloroform/methanol/acetic acid (65:25:8, v/v/v) (Haselier et al., 2010) along with PG and CL (Sigma–Aldrich) as standards and radioactive spots were visualized by Imaging Plate (Fuji Film) and BAS-2500 (GE Healthcare). Unlabeled PG and CL were stained with 0.01% primuline in 80% (v/v) acetone and detected under UV light.

### RNA Extraction and cDNA Synthesis

RNA extraction and cDNA synthesis were performed as previously described (Hung et al., 2013).

### Quantitative RT-PCR

Quantitative RT-PCR analysis involved the ABI 7500 Real Time PCR System (Applied Biosystems) with the specific oligonucleotide primer sets, CH955 and CH956, CH957 and CH958, and CH531 and CH532, for *CrCLS1, CRD1*, and *ACT1*, respectively. Gene expression was normalized to that of *ACT1*. Data were averaged by three technical replicates in the same run and three biological replicates in separate runs. The primer sequences are described in Supplementary Table S2.

## Results

### Sequence Analysis of CrCLS1

**FIGURE 1:**
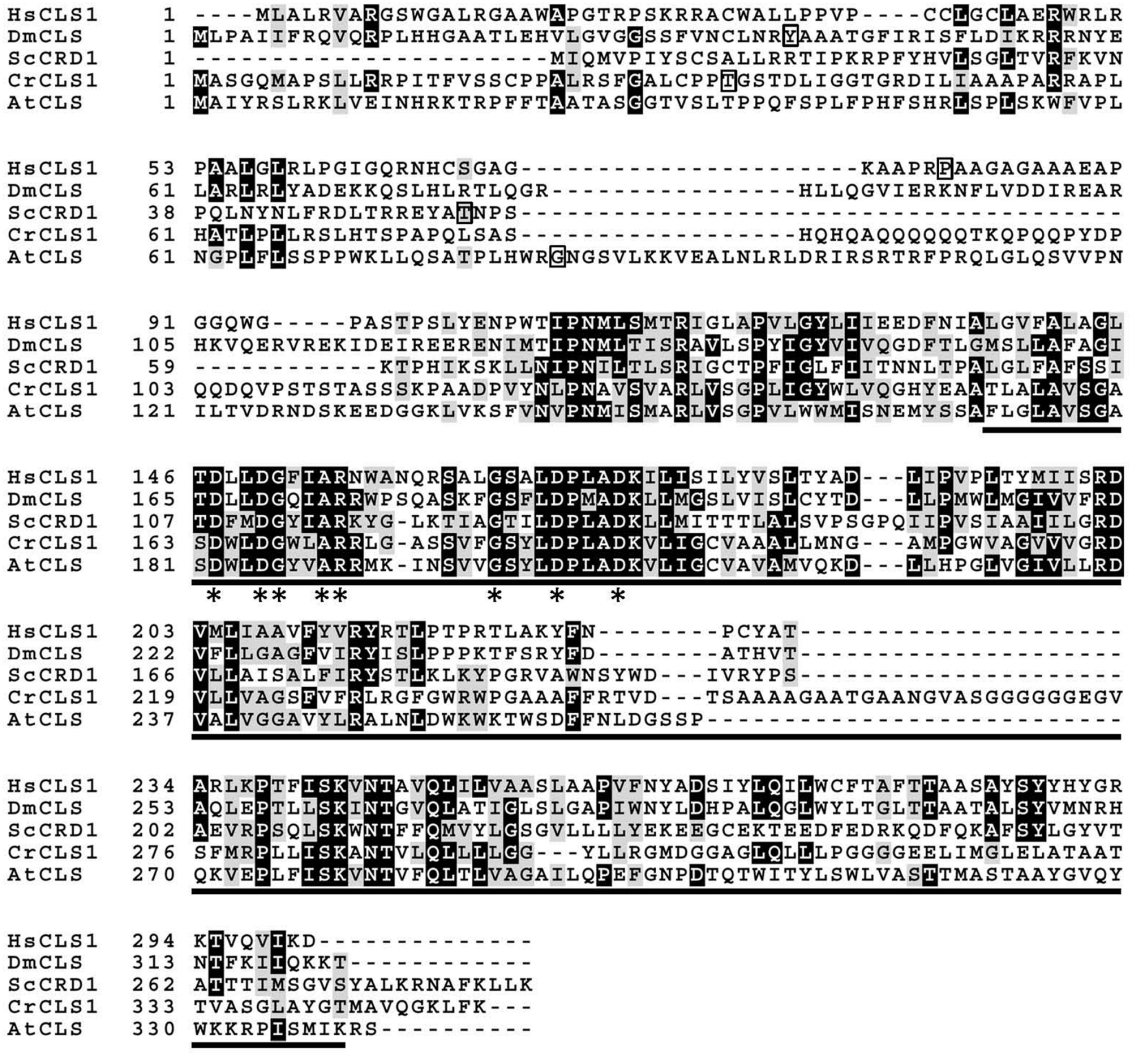
**Multiple amino acid sequence alignment of *Chlamydomonas reinhardtii* CLS1 (CrCLS1) with other known CLSs, *Saccharomyces cerevisiae* CRD1 (ScCRD1), *Homo sapiens* CLS1 (HsCLS1), *Arabidopsis thaliana* CLS (AtCLS), and *Drosophila melanogaster* CLS (DmCLS).** The region conserved among proteins containing a CDP-OH-P motif (PF01066.9) is underlined. Asterisks indicate the amino acid residues conserved in all sequences of proteins with the CDP-OH-P motif. Square frames indicate the terminal amino acid residues of the predicted cleavage site of putative N-terminal mitochondrial targeting sequence.

To compare the amino acid sequence similarity of the putative CrCLS1 with other known CLSs in different organisms, the deduced amino acid sequence of CrCLS1 was compared with those of *S. cerevisiae* CRD1, *Homo sapiens* CLS1, *A. thaliana* CLS, and *Drosophila melanogaster* CLS, which are functionally characterized CLS (**Figure 1**) (Tuller et al., 1998; Katayama et al., 2004; Chen et al., 2006; Acehan et al., 2011). In **Figure 1**, the region containing the CDP-OH-P motif D(X)_2_DG(X)_2_AR(X)_8-9_G(X)_3_D(X)_3_D is underlined and asterisks indicate the conserved eight amino acid residues. All eight amino acids were conserved in CrCLS1, which suggests that *CrCLS1* encodes a functional CLS. In addition, CrCLS1 contained a putative N-terminal mitochondrial targeting sequence predicted by the subcellular localization program MitoProtII, suggesting a possible localization of CrCLS1 in mitochondria, where CL is exclusively localized.

### Complementation of *pgsA* by *CrCLS1*

**FIGURE 2:**
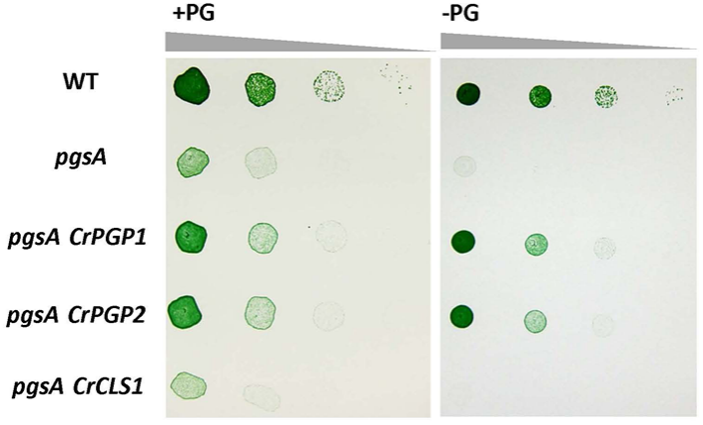
**Heterologous complementation of *Synechocystis* sp. PCC 6803 *pgsA* mutant by *CrPGP1, CrPGP2* and *CrCLS1*.** Growth of the wild type, *pgsA, pgsA CrPGP1, pgsA CrPGP2*, and *pgsA CrCLS1* were compared on solid BG-11 media with or without PG supplementation. Spotting involved serial 10-fold dilution from left to right starting at OD_730_ of 0.05, with 5 μl each spotted onto a BG-11 agar plate with or without 20 μM PG and incubated under 50∼60 μmol photons m^-2^ s^-1^ for 5 days at 30°C. Images are representative of three biological replicates.

To examine whether CrCLS1 functions as PGPS, we transformed CrCLS1 into the *pgsA* mutant of *Synechocystis* sp. PCC 6803, which abolishes PGPS activity and thus requires exogenous supplementation of PG for growth (Hagio et al., 2000). As shown in **Figure 2**, whereas the *CrPGP1* and *CrPGP2* functionally complemented the lethal phenotype of the *pgsA* mutant as reported previously (Hung et al., 2015a), *CrCLS1* failed to complement the growth phenotype, showing the rescued growth only in the presence of PG. Therefore, CrCLS1 does not function as a PGPS *in vivo* in *Synechocystis* sp. PCC 6803.

### Recovery of Growth Defect in the Δ*crd1* Mutant Complemented by *CrCLS1*

**FIGURE 3:**
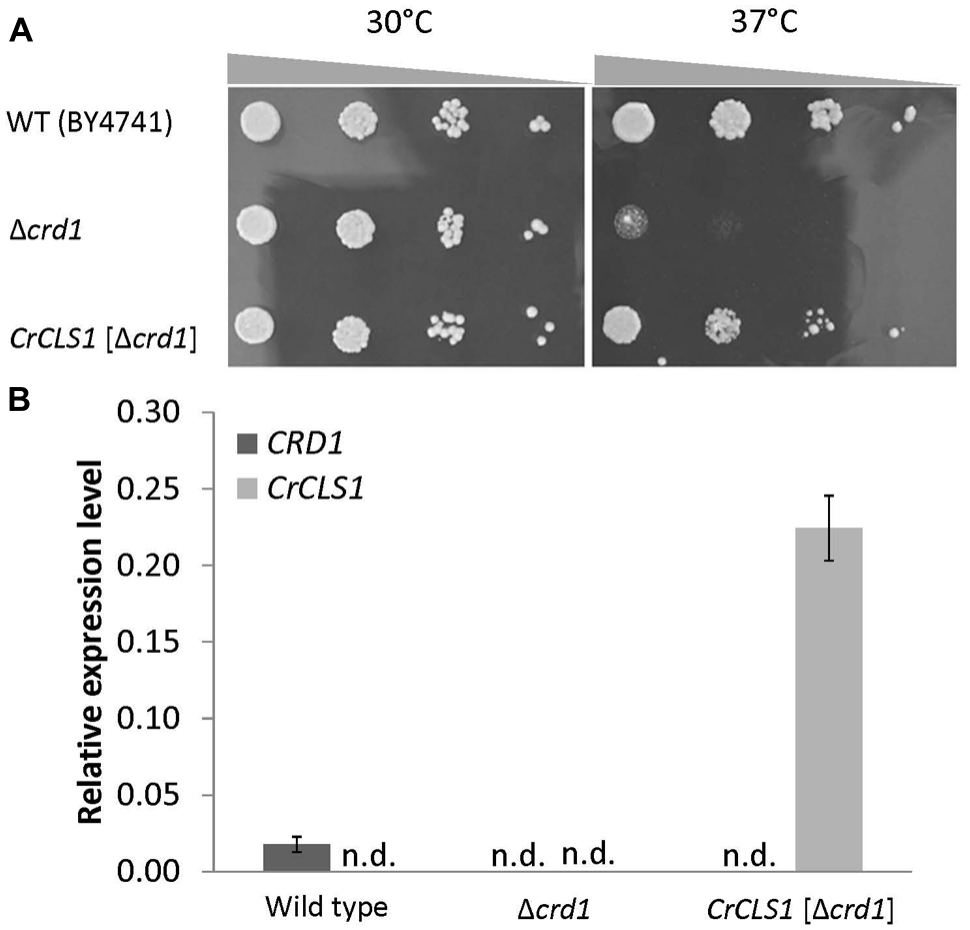
**Heterologous complementation of temperature-sensitive growth defect in the *S. cerevisiae Δcrd1* mutant with *C. reinhardtii CLS1.* (A)** Culture of wild type, Δ*crd1* mutant and Δ*crd1* mutant harboring *CrCLS1* involved serial 10-fold dilution from left to right starting at OD_600_ of 0.1; 5 μL each was spotted onto YPD media and incubated for 2 days at 30°C or 37°C. Images are representative of 3 replicates. **(B)** Relative expression of *CRD1* and *CrCLS1* in wild type, *Δcrd1* and *Δcrd1 CrCLS1*. Levels are normalized to that of *ACT1*. Data are mean ± SD from three biological replicates. n.d., not detected.

To investigate whether *CrCLS1* encodes a functional CLS, we performed a heterologous complementation assay with the *S. cerevisiae* Δ*crd1* mutant, because *Synechocystis* sp. PCC 6803 does not contain CL and no other CLS mutant is known in algae. As previously reported, Crd1p has CLS activity and Δ*crd1* mutant cells show a temperature-sensitive growth defect, severe at 37°C but not at 30°C (Jiang et al., 1999). The temperature-sensitive phenotype of Δ*crd1* mutant cells was rescued by heterologous complementation of *HsCLS1* (Houtkooper et al., 2006), so we used this approach to investigate the function of CrCLS1. We cloned the ORF of *CrCLS1* into a yeast shuttle vector and transformed it into Δ*crd1* mutant cells. The Δ*crd1* mutant harboring *CrCLS1* fully recovered cell growth at 37°C, whereas the Δ*crd1* mutant alone showed a growth defect at this temperature (**Figure 3A**). Therefore, *CrCLS1* complemented the temperature-sensitive phenotype of Δ*crd1*, which suggests that *CrCLS1* encodes a functional CLS of *C. reinhardtii*.

### Expression of *CrCLS1*

To investigate whether *CrCLS1* is appropriately expressed in the Δ*crd1* mutant, we analyzed the gene expression of *CrCLS1* in the Δ*crd1* mutant harboring *CrCLS1*. The relative gene expression of *CrCLS1* was 12.6-fold higher in the Δ*crd1* mutant harboring *CrCLS1* than *CRD1* in the wild type (**Figure 3B**). Thus, *CrCLS1* is sufficiently expressed in Δ*crd1* mutant cells, which supports the functional complementation shown in **Figure 3A**.

### Effect of Different Carbon Sources on the Growth of Δ*crd1* Mutant Complemented by *CrCLS1*

**FIGURE 4:**
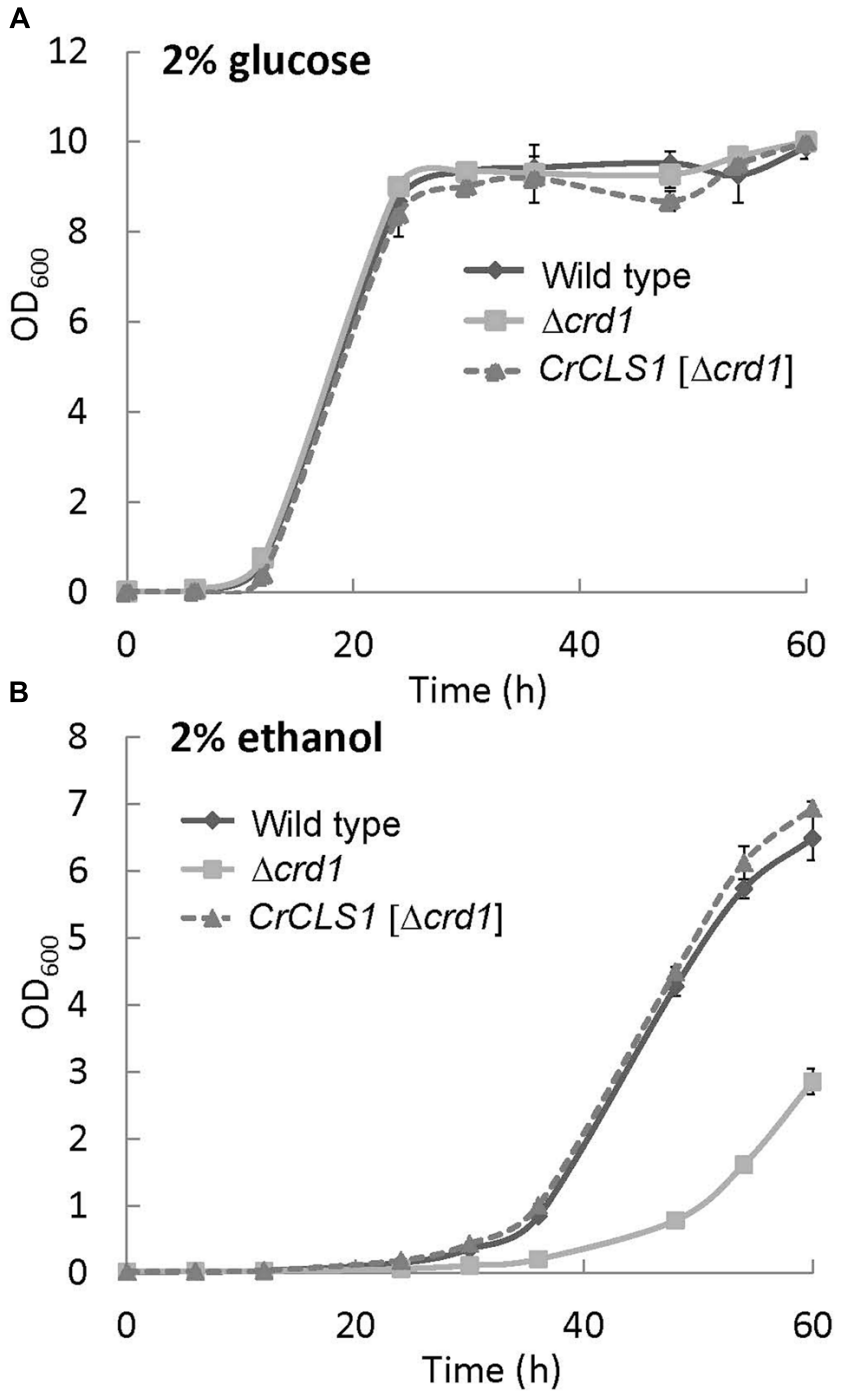
**Growth of wild type, *Δcrd1* and *Δcrd1 CrCLS1* with different carbon sources.** Cells were grown in synthetic complete medium supplemented with 2% glucose **(A)** or 2% ethanol **(B)**. Cell growth was started at OD_600_ of 0.01 at 30°C. Data are mean ± SD from three biological replicates.

A previous study showed that the growth of the Δ*crd1* mutant under aerobic conditions was affected with ethanol used as the sole carbon source (Tuller et al., 1998). To investigate whether the Δ*crd1* mutant harboring *CrCLS1* rescued the growth defect under this condition, cells were grown in synthetic complete medium supplemented with 2% glucose or 2% ethanol as the sole carbon source. The growth rates of both the *Δcrd1* mutant and *Δcrd1* harboring *CrCLS1* were indistinguishable from that of wild type in 2% glucose medium (**Figure 4A**). However, with 2% ethanol medium, the *Δcrd1* mutant harboring *CrCLS1* fully restored the growth phenotype to that of the wild type, whereas the *Δcrd1* mutant showed growth retardation, as reported (Tuller et al., 1998) (**Figure 4B**). Therefore, *CrCLS1* could complement the growth defect of the *Δcrd1* mutant with ethanol supplementation as the carbon source.

### Lipid Contents of the *Δcrd1* Mutant Complemented by *CrCLS1*

**FIGURE 5:**
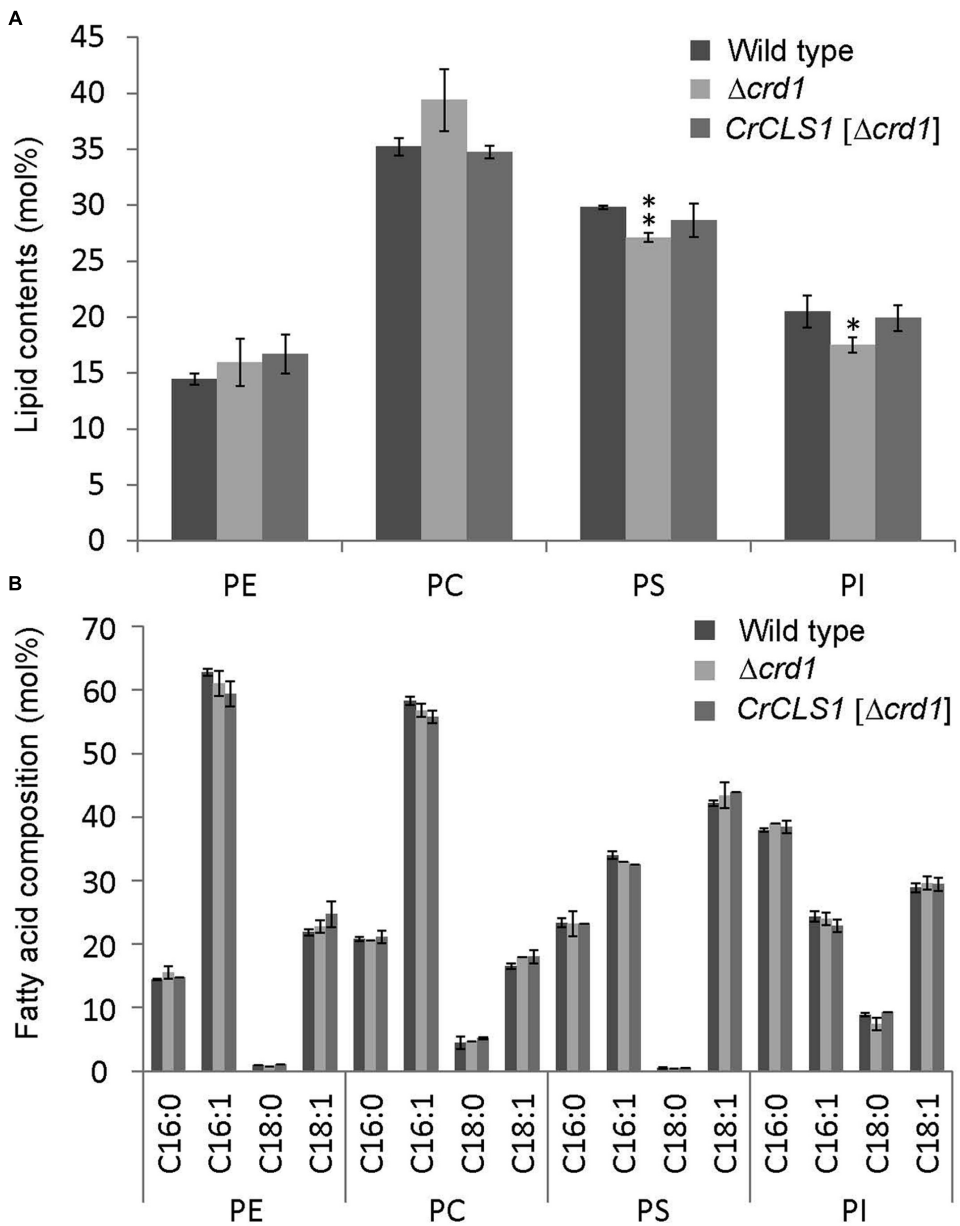
**Phospholipid content **(A)** and fatty acid composition **(B)** of wild type, *Δcrd1* and *Δcrd1 CrCLS1*.** Total lipids were extracted from cells grown to stationary phase and separated by 2D thin-layer chromatography; content of phospholipids was quantified by gas chromatography. Data are mean ± SD from three biological replicates. Asterisks indicate statistical significance by Student’s *t*-test (^∗^*P* < 0.05, ^∗∗^*P* < 0.001). PC, phosphatidylcholine; PE, phosphatidylethanolamine; PI, phosphatidylinositol; PS, phosphatidylserine.

The phospholipid profiles of the Δ*crd1* mutant were previously analyzed by radiolabeling (Tuller et al., 1998) or mass spectrometry (Zhang et al., 2003). However, whether the Δ*crd1* mutant alters the composition of major membrane phospholipid classes remained unclear. To investigate whether the complementation of the growth defect observed in **Figures 3** and **4** is associated with lipid compositional change, we analyzed the major phospholipid composition of these strains. The Δ*crd1* mutant showed an increase in PC content and decrease in PS and PI contents as compared with the wild type (**Figure 5A**). In the Δ*crd1* mutant harboring *CrCLS1*, phospholipid composition was restored to a level similar to that of the wild type. The fatty acid composition of PE, PC, and PI was similar among the three strains (**Figure 5B**). Thus, *CrCLS1* encodes a functional CLS that complements lipid compositional changes in the Δ*crd1* mutant.

### Enzyme Activity of *CrCLS1* Expressed in the Δ*crd1*

**FIGURE 6:**
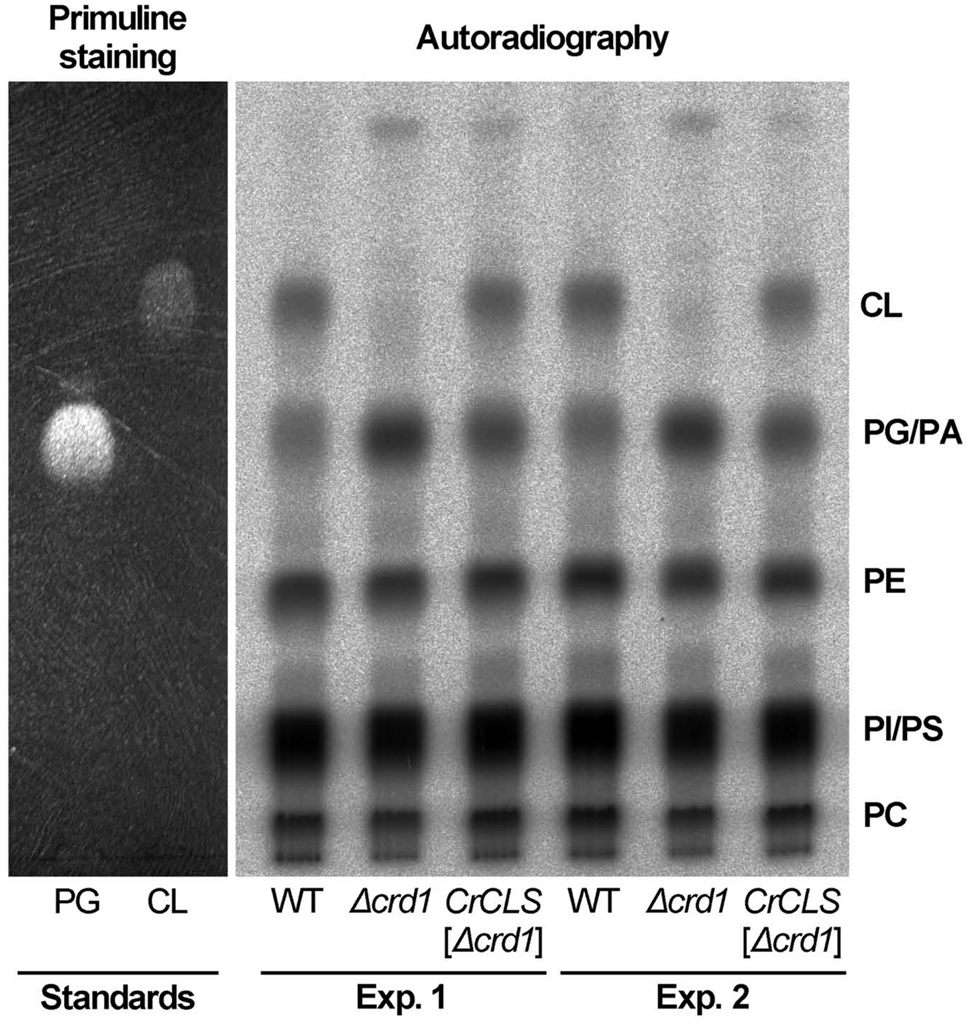
***CrCLS* complemented cardiolipin synthesis in *Δcrd1* cells.** Wild type (WT), *Δcrd1* or *Δcrd1 CrCLS* cells were grown in the presence of [^32^P]-phosphate for 8 h at room temperature. Radioactivity incorporated into phospholipids was determined by TLC analysis and autoradiography, with unlabeled PG and CL visualized by primuline staining as standards. Two biologically independent experiments (Experiments 1 and 2) were shown. PE, phosphatidylethanolamine; PC, phosphatidylcholine; PI, phosphatidylinositol; PS, phosphatidylserine; PA, phosphatidic acid.

To investigate whether *CrCLS1* encodes a functional CLS to restore the CL synthesis defect in the *Δcrd1*, we performed radiolabeling assay to analyze CLS activity. As shown in **Figure 6**, the Δ*crd1* mutant harboring *CrCLS1* recovered radiolabeled spot that co-migrates with the commercial standard of CL, which is present in wild type but absent in the Δ*crd1* mutant, demonstrating that the activity of CLS was recovered in the mutant harboring *CrCLS1*. Thus, *CrCLS1* encodes a functional CLS that complements CL synthesis defect of Δ*crd1* mutant.

## Discussion

Present study reported identification of a *CLS* gene in *C. reinhardtii, CrCLS1*, and examined its *in vivo* function by heterologous complementation of *pgsA*, a PGPS mutant of *Synechocystis* sp. PCC 6803, and *Δcrd1*, a CLS mutant of *S. cerevisiae*. Whereas *CrCLS1* did not complement the growth phenotype of *pgsA*, it rescued the temperature-sensitive growth phenotype, growth profile with different carbon sources, phospholipid composition and enzyme activity of *Δcrd1* of *S. cerevisiae*. These results suggest that *CrCLS1* is a functional gene for CLS of *C. reinhardtii* as the first identified algal CLS, which is functionally incompatible with PGPS despite their sequence homology.

Physiological roles of CrCLS1 in *C. reinhardtii* are not reported yet; however, several transcriptomic studies have shown gene expression profiles in response to environmental stresses. For example, expression of *CrCLS1* is down-regulated in response to the deprivation of iron (Urzica et al., 2013) and nitrogen (Goodenough et al., 2014). Conversely, an upregulation is seen by copper deficiency (Castruita et al., 2011) and singlet oxygen stress (Wakao et al., 2014). These data suggest possible roles of CrCLS1 in adaptation to circumvent environmental stresses.

Given that both PGPS and CLS belong to the CDP-alcohol phosphotransferase family and the relevant CDP-OH-P motifs are closely related (Katayama et al., 2004), what defines substrate specificity of these enzymes?

**FIGURE 7:**
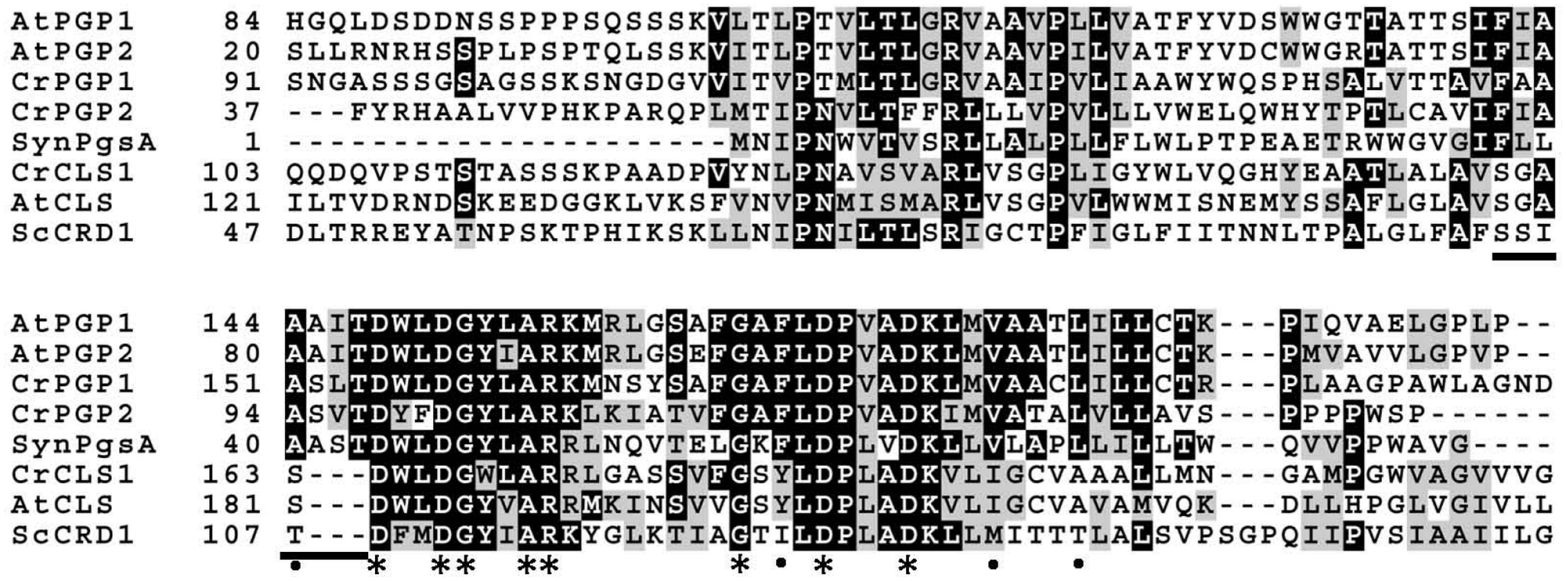
**Multiple amino acid sequence alignment of the core motif of CDP-OH-P between CLSs and the PGPSs.** Asterisks indicate the amino acid residues conserved among proteins with the CDP-OH-P motif, and dots indicate those conserved only among PGPSs. The amino acid residues present among PGPS but not among CLS are underlined. CrCLS1, *C. reinhardtii* CLS1; ScCRD1, *S. cerevisiae* CRD1; AtCLS, *A. thaliana* CLS; CrPGP1, *C. reinhardtii* PGP1; CrPGP2, *C. reinhardtii* PGP2; AtPGP1, *A. thaliana* PGP1; AtPGP2, *A. thaliana* PGP2; SynPgsA, *Synechocystis* sp. PCC 6803 PgsA.

Recently, structural basis for catalysis in a CDP-alcohol phosphotransferase was revealed by crystallographic analysis (Sciara et al., 2014). According to this structure, conserved amino acid residues in the CDP-OH-P motif are associated with CDP-DAG. Since CDP-DAG is the common substrate between CLS and PGPS, this study suggests that an additional motif recognizes the other substrate (PG for CLS; glycerol 3-phosphate for PGPS). We aligned the amino acid sequences of core CDP-OH-P motif among three CLSs (*C. reinhardtii* cardiolipin synthase 1, CrCLS1; *S. cerevisiae* CRD1, ScCRD1; *A. thaliana* CLS, AtCLS) and five PGPSs (*C. reinhardtii* PGP1, CrPGP1; *C. reinhardtii* PGP2, CrPGP2; *A. thaliana* PGP1, AtPGP1; *A. thaliana* PGP2, AtPGP2; *Synechocystis* sp. PCC 6803 PgsA, SynPgsA) (**Figure 7**). While the eight amino acid residues of the core CDP-OH-P motif D(X)_2_DG(X)_2_AR(X)_8-9_G(X)_3_D(X)_3_D indicated by asterisks in **Figure 7** were conserved between the PGPS and CLS, we noted that seven amino acids (FxxAxxT) immediately before the core CDP-OH-P motif were highly conserved among PGPSs but not CLSs (underlined in **Figure 7**). In addition, we found additional four amino acid residues that were conserved among PGPS but not in CLS (indicated by dots in **Figure 7**). It is possible that these additional residues may define the substrate specificity between PGPS and CLS. Detailed structural analysis as well as enzymatic characterization of these residues are anticipated to experimentally validate this proposal.

## Conclusion

We suggest functional specificity of CLS by the identification and characterization of a CLS, CrCLS1, in *C. reinhardtii*.

## Author Contributions

KK, HW, and YN conceived research. KK and C-HH performed experiments and analyzed data. All authors wrote and commented on the manuscript and approved the contents.

## Conflict of Interest Statement

The authors declare that the research was conducted in the absence of any commercial or financial relationships that could be construed as a potential conflict of interest.

## Footnotes

1 http://www.genome.jp/tools/clustalw/

2 http://ihg.gsf.de/ihg/mitoprot.html

## Abbreviations

CDP: cytidine 5′-diphosphate
CL: cardiolipin
CLS: cardiolipin synthase
PA: phosphatidic acid
PC: phosphatidylcholine
PE: phosphatidylethanolamine
PG: phosphatidylglycerol
PGP: phosphatidylglycerol phosphate
PGPP: phosphatidylglycerophosphate phosphatase
PGPS: phosphatidylglycerophosphate synthase
PI: phosphatidylinositol
PS: phosphatidylserine
