# Supplementary material for: Functional Specificity of Cardiolipin Synthase Revealed by the Identification of a Cardiolipin Synthase CrCLS1 in Chlamydomonas reinhardtii
Source: Front Microbiol. 2016 Jan 12;6:1542. doi: 10.3389/fmicb.2015.01542 (PMC4709463; doi:10.3389/fmicb.2015.01542)
Supplement: Supplementary file 1 [file Table_1.DOCX]

**Functional specificity between phosphatidylglycerophosphate synthase and cardiolipin synthase revealed by the isolation and characterization of a cardiolipin synthase CrCLS1 in *Chlamydomonas reinhardtii***

Chun-Hsien Hung^1^, Koichi Kobayashi^2^, Hajime Wada^2,3^, Yuki Nakamura^1,4*^

^1^Institute of Plant and Microbial Biology, Academia Sinica, Taipei, Taiwan.

^2^ Department of Life Sciences, Graduate School of Arts and Sciences, The University of Tokyo, Komaba 3-8-1, Meguro-ku, Tokyo 153-8902, Japan.

^3^ CREST , JST, 4-8-1 Honcho, Kawaguchi, Saitama 332-0012, Japan.

^4^PRESTO, JST, 4-8-1 Honcho, Kawaguchi, Saitama 332-0012, Japan.

*Corresponding: Yuki Nakamura, Institute of Plant and Microbial Biology, Academia Sinica, 128 sec.2 Academia Rd., Nankang, Taipei 11529, Taiwan. [nakamura@gate.sinica.edu.tw](mailto:nakamura@gate.sinica.edu.tw)

**Supplementary Tables**

| **Supplemental Table 1 List of strains used in this study.** | | |
| --- | --- | --- |
| **Strain** | **Genotype** | **Source** |
| BY4741 | *MATα, his3*Δ*0 leu2*Δ*0 met15*Δ*0 ura3*Δ*0* | - |
| ∆*crd1* | *gep4*∆::*KANMX,* BY4741 | Thermo scientific |
| CHY035 | pCH078, BY4741 | (Hung et al. 2015b) |
| CHY135 | pCH178, *crd1∆::KANMX*, BY4741 | This work |
| CHY136 | pCH078, *crd1∆::KANMX*, BY4741 | This work |
| *Synechocystis* sp. PCC 6803 | Wild type | - |
| *pgsA* | *pgsA*::Km^R^ | (Hagio et al. 2000) |
| CHS004 | ∆*pgsA* *slr2031*∆::*CrPGP1* | (Hung et al. 2015a) |
| CHS005 | ∆*pgsA* *slr2031*∆::*CrPGP2* | (Hung et al. 2015a) |
| CHS006 | ∆*pgsA* *slr2031*∆::*CrCLS1* | This work |
| CC-503 | cw92 mt+ | Chlamydomonas Resource Center |
