# Supplementary material for: Functional Specificity of Cardiolipin Synthase Revealed by the Identification of a Cardiolipin Synthase CrCLS1 in Chlamydomonas reinhardtii
Source: Front Microbiol. 2016 Jan 12;6:1542. doi: 10.3389/fmicb.2015.01542 (PMC4709463; doi:10.3389/fmicb.2015.01542)
Supplement: Supplementary file 2 [file Table_2.DOCX]

| **Supplemental Table 2 List of oligonucleotides used in this study.** | |
| --- | --- |
| Name | Sequences (5’ to 3’) |
| CH227 | CACCATGGCATCTGGGCAGATGGCGCCAT |
| CH228 | CTACTTGAACAGCTTGCCCTGCACC |
| CH776 | GGAATTCCATATGGAATTCCATGGCATCTGGGCAGATGGCGCCA |
| CH777 | TAGGTTAACCTACTTGAACAGCTTGCCCTGCAC |
| CH831 | GGATCTAGAATGGCATCTGGGCAGATGGCGCCA |
| CH832 | TAGGAATTCCTACTTGAACAGCTTGCCCTGCAC |
| CH531 | GACGCTCCTCGTGCTGTCTT |
| CH532 | ACCGTGTTCAATTGGGTAACG |
| CH955 | CGATCACGTTCGTGTCCAGTT |
| CH956 | GCCTGTGCCTCCGATGAG |
| CH957 | TGCATTTTCCAGCATCACTGA |
| CH958 | TGAGGGCCGGATGGTACA |
|  |  |
