# Supplementary material for: Functional Specificity of Cardiolipin Synthase Revealed by the Identification of a Cardiolipin Synthase CrCLS1 in Chlamydomonas reinhardtii
Source: Front Microbiol. 2016 Jan 12;6:1542. doi: 10.3389/fmicb.2015.01542 (PMC4709463; doi:10.3389/fmicb.2015.01542)
Supplement: Supplementary file 3 [file Table_3.DOCX]

| **Supplemental Table 3 List of plasmids used in this study.** | | | | | |  |
| --- | --- | --- | --- | --- | --- | --- |
| Plasmid | Encoded gene | Promoter | Vector | Source | |  |
| pCH069 | *CrCLS1* | - | pENTR/D-TOPO | | Invitrogen | |
| pCH078 | - | *GPD* | pYES2/NTA | | (Hung et al. 2013) | |
| pCH151 | - | *psbA2* | pTCP2031V | | (Satoh et al. 2001) | |
| pCH158 | *CrCLS1* | *psbA2* | pTCP2031V | | This work | |
| pCH178 | *CrCLS1* | *GPD* | pYES2/NTA | | This work | |
